# Cybersecurity of Critical Infrastructures: Challenges and Solutions

**DOI:** 10.3390/s22145105

**Published:** 2022-07-07

**Authors:** Leandros Maglaras, Helge Janicke, Mohamed Amine Ferrag

**Affiliations:** 1 School of Computer Science and Informatics, De Montfort University, Leicester LE1 9BH, UK; 2 Cyber Security Cooperative Research Centre, Edith Cowan University, Perth 6027, Australia; helge.janicke@cybersecuritycrc.org.au; 3 Department of Computer Science, Guelma University, Guelma 24000, Algeria; ferrag.mohamedamine@univ-guelma.dz

People’s lives are becoming more and more dependent on information and computer technology. This is accomplished by the enormous benefits that the ICT offers for everyday life. Digital technology creates an avenue for communication and networking, which is characterized by the exchange of data, some of which are considered sensitive or private. There have been many reports recently of data being hijacked or leaked, often for malicious purposes. Maintaining security and privacy of information and systems has become a herculean task. It is therefore imperative to understand how an individual’s or organization’s personal data can be protected. Moreover, critical infrastructures are vital resources for the public safety, economic well-being and national security.

The major target of cyber attacks can be a country’s Critical National Infrastructures (CNIs) like ports, hospitals, water, gas or electricity producers, that use and rely on Industrial Control Systems but are affected by threats to any part of the supply chain. Cyber attacks are increasing at rate and pace, forming a major trend. The widespread use of computers and the Internet, coupled with the threat of activities of cyber criminals, has made it necessary to pay more attention to the detection or improve the technologies behind information security. The rapid reliance on cloud-based data storage and third-party technologies makes it difficult for industries to provide security for their data systems. Cyber attacks against critical systems are now common and recognized as one of the greatest risks facing today’s world [1].

This editorial presents the manuscripts accepted, after a careful peer-review process, for publication in the topic “Cyber Security and Critical Infrastructures” of the MDPI journals Applied Sciences, Electronics, Future Internet, Sensors and Smart Cities. The first volume includes sixteen articles: one editorial article, fifteen original research papers describing current challenges, innovative solutions, and real-world experiences involving critical infrastructures and one review paper focusing on the security and privacy challenges on Cloud, Edge, and Fog computing.

Many companies have recently decided to use cloud, edge and fog computing in order to achieve high storage capacity and efficient scalability. The work presented in [2] mainly focuses on how security in Cloud, Edge, and Fog Computing systems is achieved and how users’ privacy can be protected from attackers. The authors mention that there is a huge potential for vulnerabilities in security and privacy of such system. One good way of screening systems for possible vulnerabilities is by performing auditing of the systems based on security standards.

The recent EU Directive on security of network and information systems (the NIS Directive) has identified transport as one of the critical sectors that need to be secured in a European level. Smart cars is changing the transport landscape by introducing new capabilities along with new threats. Focusing on vehicle security, the authors in [3] examine the bit-level CAN bus reverse framework using a multiple linear regression model. The increasingly diverse features in today’s vehicles offer drivers and passengers a more relaxed driving experience and greater convenience along with new security threats. The reverse capability of the proposed system can help automotive security researchers to describe vehicle behavior using CAN messages when DBC files are not available.

Vulnerabilities in computer programs have always been a serious threat to software security, which may cause denial of service, information leakage and other attacks. The authors in [4] propose a new framework of fuzzy testing sample generation called CVDF DYNAMIC. which consists of three parts: Sample generation based on a genetic algorithm, sample generation based on a bi-LSTM neural network and sample reduction based on a heuristic genetic algorithm.

The transformation of cities into smart cities is on the rise. Through the use of innovative technologies such as the Internet of Things (IoT) and cyber–physical systems (CPS) that are connected through networks, smart cities offer better services to the citizens. The authors in propose a novel machine learning solution for threat detection in a smart city [5].The proposed hybrid Deep learning model that consists of QRNN and CNN improves cyber threat analysis accuracy, loweres False Postitive rate, and provides real-time analysis. The authors evaluated the proposed model on two datasets that were simulated to represent a realistic IoT environment and proved its superiority.

The next article in this collection [6] proposes a novel framework for few-shot network intrusion detection. Based on the fact that DL methods have been widely successful as network-based IDSs but require sizeable volumes of datasets which are not always feasible, the authors focus on few-shot solutions. Their proposed method is suitable for detecting specific classes of attacks. This model could be very helpful for deploying novel IDSs for Industrial Control Systems, which are the core of Critical Infrastructures, where there is a general lack of datasets.

In [7] the authors propose a novel reversible data hiding (RDH) scheme that can be applied to either remote medical diagnosis or even military secret transmission. The authors utilize a trained multi-layer perception neural network in order to be able to predict pixel values and then combining those with prediction error expansion techniques (PEE) to achieve (RDH). The proposed method although efficient is very time consuming and the authors propose in the future to implement novel solution to improve this aspect.

Focusing on Industrial components that are the main parts of critical infrastructures the authors in [8] propose a model for vulnerability analysis through the their entire life-cycle. The model can Identify the root causes and nature of vulnerabilities for the industrial components. This information is useful extracting new requirements and test cases, support the prioritization of patching and track vulnerabilities during the whole life-cycle of industrial components. The proposed model is applicable to existing systems and can be a good source of information for defining patching, training and security needs.

Android mobile devices are becoming the targets of several attacks nowadays since they support many of the everyday digital needs of the users. Since many sensitive applications are offered in these smart devices, like e-banking, adversaries have launched a number of new attacks. IoT enhances the power of malicious entities or people to perform attacks on critical systems or services. A lot of connected devices additionally mean a bigger attack surface for attacks and greater risk. Hackers using infected devices can generate many frequent, organized and complex malicious attacks. The authors in [9] propose novel IDS for malware in android devices combining several machine learning techniques. The proposed classifiers achieved good accuracy outperforming existing state-of-the-art models.

Having identified a lack of studies related to security in microservices architecture and especially for for authentication and authorization to such systems, the authors in [10] perform an analysis about this open issue. Microservices can increase scalability, availability and reliability of the system but come with an increase in the attack surface and new threats in the communication between them. Since microservices can become an integral part of critical systems, a thorough research on the attacks and defence against them is crucial. The article concludes that several existing solutions can be applied to make the systems robust but also novel methods need to be proposed that are tailored to the new architectures.

In another article that deals with machine learning as a defence mechanism for smart systems, the authors in [11] focus on the correct feature selection. Feature selection is the process of correctly identifying those features that help the machine learning algorithm be robust against an adversary. The article proposes a smart feature selection process and a novel feature engineering process which are proven to be more precise in terms of manipulated data while maintaining good results on clean data. The proposed solutions can be easily adopted in real environments in order to deal with sophisticated attacks against critical infrastructures.

Information Security Awareness Training is used to raise awareness of the users against cyber attacks and help them build a responsible behavior. In [12] the authors try to answer the question whether game-based training and Context-Based Micro-Training (CBMT) can help users correctly identify phishing against legitimate emails. IN order to answer this question the authors conducted a simulated experiment with 41 participants and the results showed that both methods managed to improve user behavior in relation to phishing emails. The paper concludes that training is a strong tool against cyber attacks but must be combined with other security solutions.

A vital challenge faced nowadays by federal and business decision-makers for choosing cost-efficient mitigations to scale back risks from supply chain attacks, particularly those from adversarial attacks that are complex, hard to detect and can lead to severe consequences. Focusing on adversarial attacks and how these can alter the performance of AI based detection systems, the authors in [13] propose a novel robust solution. Their proposed model was evaluated in both Enterprise and Internet of Things (IoT) networks and is proven to be efficient against adversarial classification attacks and adversarial training attacks.

There are many reasons why it’s vital to know what users can perceive as believable. It is crucial for service suppliers to grasp their vulnerabilities so as to assess their exposure to risks and also the associated problems. moreover, recognizing what the vulnerabilities are interprets into knowing from wherever the attacks are likely to come which leads for appropriate technical security measures to be deployed to protect against attacks. In [14] the authors present a solution that combines deep neural network and frequency domain pre-processing in order to detect images with embedded spam in social networks. The proposed method is proven to be superior against state-of-the-art detection models in terms of detection accuracy and efficiency. One of the major contributions of the authors is the creation of a novel dataset that contains images with embedded spam, which will be expanded in the near future.

Finding the correct sources that include vital information about securing critical systems is very important. Unfortunately, the lack of a fully functioning semantic web or text-based solutions to formalize security data sources limits the exploitation of existing cyber intelligence data sources. In [15] the authors aim to empower ontology-based cyber intelligence solutions by presenting a security ontology framework for storing data in an ontology from various textual data sources, supporting knowledge traceability and evaluating relationships between different security documents.

Ransomware has become one of the major threats against critical systems the latest years. The recent report from ENISA has ranked ransomware attacks first in terms of severity and frequency. Current solutions against ransomware do not cover all possible risks of data loss. In this article [16], the authors try to address this aspect and provide an effective solution that ensures efficient recovery of XML documents after ransomware attacks.

## Data Availability

Not applicable.
